# Quantitative aerobiologic analysis of an influenza human challenge‐transmission trial

**DOI:** 10.1111/ina.12701

**Published:** 2020-06-15

**Authors:** Paul Jacob Bueno de Mesquita, Catherine J. Noakes, Donald K. Milton

**Affiliations:** ^1^ Maryland Institute for Applied Environmental Health University of Maryland College Park Maryland USA; ^2^ School of Civil Engineering University of Leeds Leeds UK

**Keywords:** aerosols, airborne infection, Influenza virus, rebreathed air, risk assessment, transmission

## Abstract

Despite evidence that airborne transmission contributes to influenza epidemics, limited knowledge of the infectiousness of human influenza cases hinders pandemic preparedness. We used airborne viral source strength and indoor CO_2_ monitoring from the largest human influenza challenge‐transmission trial (EMIT: Evaluating Modes of Influenza Transmission, ClinicalTrials.gov number NCT01710111) to compute an airborne infectious dose generation rate *q* = 0.11 (95% CI 0.088, 0.12)/h and calculate the quantity of airborne virus per infectious dose *σ* = 1.4E + 5 RNA copies/quantum (95% CI 9.9E + 4, 1.8E + 5). We then compared these calculated values to available data on influenza airborne infectious dose from several previous studies, and applied the values to dormitory room environments to predict probability of transmission between roommates. Transmission risk from typical, moderately to severely symptomatic influenza cases is dramatically decreased by exposure reduction via increasing indoor air ventilation. The minority of cases who shed the most virus (ie, supershedders) may pose great risk even in well‐ventilated spaces. Our modeling method and estimated infectiousness provide a ground work for (a) epidemiologic studies of transmission in non‐experimental settings and (b) evaluation of the extent to which airborne exposure control strategies could limit transmission risk.

**Practical Implications**

- Quantitative aerobiologic analysis of the EMIT transmission trial and comparison with a proof‐of‐concept study suggests that the airborne mode may have driven transmission in these settings. The calculated airborne infectious dose generation rate and airborne RNA copies per infectious dose, and the presented method support new efforts to estimate, predict, and validate airborne infectious doses and transmission risk given knowledge of indoor air CO_2_ or ventilation and estimates of exhaled breath viral shedding rates.

## 1 INTRODUCTION

The substantial, global disease burden attributed to seasonal influenza and the threat of pandemic influenza demand improved preparedness. Each year influenza has caused up to 650 000 respiratory deaths globally, and as many as 960 000 hospitalizations and over $11 billion in economic burden in the United States.^1^, ^2^, ^3^ Efforts to develop and improve vaccines (related to possible differential immunity stimulated by infection initiation in lung vs upper respiratory mucosa) and other prevention strategies are hindered by deficient understanding about the competing risk contributions of contact, large droplet, and droplet nuclei (ie, aerosol or airborne) transmission modes. Influenza virus has been detected in exhaled breath droplet nuclei,^4^ and there is evidence supporting airborne transmission as a driver of epidemics and severe illness.^5^, ^6^, ^7^, ^8^, ^9^, ^10^, ^11^, ^12^ Quantifying airborne risk is widely accepted as a necessary step for quelling outbreaks.

Wells postulated an airborne quantum generation rate *q* as the infectious doses generated by an infected individual during exposure with a susceptible.^13^ The Wells‐Riley equation (Equation S1) facilitates the computation of *q* with knowledge of secondary attack rate from exposure between primary cases and susceptibles and indoor ventilation rates.^14^ Rudnick and Milton's “rebreathed air” version of the Wells‐Riley equation (Equation S2) is well suited for practical exposure assessment under non‐steady state conditions through measurement of indoor and background CO_2_ levels to estimate indoor occupant exposure to exhaled breath (Appendix S1).^15^ It is based on the knowledge that (a) droplet nuclei are emitted through the exhaled breath of infectious individuals, and (b) CO_2_ contained in exhaled breath is constant at 3.8E + 4 ppm and is the predominant source of CO_2_ in buildings, a valid assumption for environments without significant combustion sources. Assuming a well‐mixed space, the rebreathed air equation uses measured CO_2_ levels to directly estimate exposure to exhaled breath that may be contaminated with a quantifiable level of infectious particles.

Despite the challenge of accurately quantifying exposure between infected and susceptible humans,^16^ a 2013 US CDC‐funded influenza transmission‐challenge trial in a controlled environment gives an observed secondary attack rate with airborne viral exposure data (EMIT; ClinicalTrials.gov number NCT01710111).^17^ The trial tested the effect of using face shields and stringent hand hygiene on transmission risk; these measures were considered to prevent droplet and contact transmission and hence isolate the airborne mode of transmission. We applied the rebreathed air equation to these data to characterize the relationship between airborne exposure and infection risk. We estimated the airborne quantum generation rate *q* for influenza H3, and we estimated *σ*, the RNA copies in fine particle exhaled breath aerosols per quantum. We compared these calculated values to available data on influenza airborne infectious dose: NIOSH exposure room air samples from EMIT, a proof‐of‐concept human challenge‐transmission study,^18^ an airliner outbreak,^19^ and symptomatic, naturally infected cases.^4^ Finally, we applied the values to CO_2_‐monitored dormitory room environments to predict transmission probability between roommates.

## 2 METHODS

### 2.1 EMIT trial design

The EMIT challenge‐transmission trial methods are described elsewhere.^17^ In brief, seronegative volunteers were randomized to viral “Donor” (N = 52), “Intervention Recipient” (N = 40), and “Control Recipient” (N = 35) groups. Intervention recipients wore face shields and observed strict hand hygiene to eliminate droplet and contact transmission routes, but still enable airborne transmission. Control Recipients wore no face shields and had normal hand hygiene, and were considered to be exposed through airborne, droplet, and contact transmission. Donors were inoculated with 0.5 mL per nostril of a suspension containing 5.5log_10_TCID_50_/mL of influenza H3/Wisconsin/67/2005 from current, good manufacturing practices. Recipients were exposed to Donors for four consecutive Study Days (~13‐16 h/d) and assessed for evidence of infection. Infection was determined by paired serology (four‐fold rises in MN or HAI assays), or by 2 days of positive qRT‐PCR tests on nasopharyngeal swabs collected between Days 1 and 6 after inoculation.

The transmission trial used three quarantine periods. Quarantines 1, 2, and 3 used five, three, and five exposure rooms, respectively, for 13 total exposure rooms. Recipients were assigned to exposure groups (EGs) on Day 1 and switched rooms each Day with their group. In an attempt to evenly distribute viral source strength, Donors assigned to EGs on Study Day 1 were reallocated, according to clinical presentation on Day 2, to new EGs where they remained through the end of exposure. Three Recipients were withdrawn from exposure due to symptom presentation to prevent a second generation of transmission; none tested positive for influenza by Sofia rapid test.

Mechanically ventilated exposure rooms were characterized prior to Quarantines to ensure similar conditions in all rooms within and between Quarantines (Appendix S5). Continuous environmental monitoring was conducted throughout each Quarantine to control indoor CO_2_ concentrations, relative humidity, and temperature to produce what were considered as favorable conditions for influenza transmission, while balancing the thermal comfort of volunteers, and the capacity of the building's mechanical ventilation system to attain stable conditions throughout the study. The exposure rooms were well‐mixed spaces confirmed by tracer gas studies.

### 2.2 Pulmonary ventilation rates assumed homogeneous

Here, we assumed the pulmonary ventilation rates of Donors and Recipients (and study monitors who also spent time in the rooms) were similar. Given that volunteers participated in similar, lightly or non‐physical activities, and never experienced severe illness, the main differences in the contribution to exhaled breath in the room would be related to baseline respiratory function, which was not likely to be substantially different between healthy, young adult volunteers.

### 2.3 Quantifying viral shedding in exhaled breath

Viral shedding into exhaled breath aerosols was collected from Donors with a Gesundheit‐II,^4^, ^20^ into “fine” (≤5 µm and >0.05 µm in diameter) and “coarse” (>5 µm) fractions. Evaluation of nasopharyngeal swabs and exhaled breath aerosols by qRT‐PCR was done in duplicate using the protocol from Yan et al.^4^ The limit of detection (detection of at least one replicate) was 500 RNA copies/sample; the limit of quantification (detection of all replicates) was 2000 RNA copies/sample.

For samples where both qRT‐PCR replicates were above the limit of detection, a replicate mean was taken from measured values. No Donors demonstrated detectable RNA in fine particle aerosols on Study Day 1 of each Quarantine, so the fine aerosol shedding period was assumed to be Study Days 2‐4. For infected Donors who ever shed into aerosols with at least one detectable qRT‐PCR replicate, aerosol shedding was imputed for replicates below detection limit and on instances during Study Days 2‐4 where some samples were not collected. For each of these Donors *i,* on each Study Day *j,* the rate of RNA copies shed into fine aerosols/h (CPH) is defined by *V*_ij_ and imputed by ${\hat{V}}_{\mathrm{ij}}$ (Equation 1) from Tobit regression (SAS Proc NLMIXED)^21^ with intercept *β*_0_ fixed effects of self‐reported cough symptom *β*_1_ and Study Day *β*_2_, random effect intercept *b*_0_ and random effect of Donor *b*_1_, using 100 quadrature points. Tobit regression parameter coefficients were taken from generalized linear models (SAS Proc GENMOD) with fixed effects of self‐reported cough symptom and Study Day. Self‐reported cough symptoms were collected thrice daily on an ordinal scale 0‐3 (3 most severe) and daily averages were used in regression models.

(1) $${\hat{V}}_{\mathrm{ij}}∼\beta_{0}+\beta_{1}*{\text{cough}}_{\mathrm{ij}}+\beta_{2}*\text{study}\;{\text{day}}_{\mathrm{ij}}+b_{0}+b_{1}*i.$$

More information on viral shedding estimates is presented in Appendix S3.

### 2.4 Linking *q* with measurable airborne virus

One transmission event was observed in Quarantine 2 yielding an overall secondary attack rate (SAR) of 1/75 (1.33%) from exposure to viral Donors. Nguyen‐Van‐Tam and colleagues suggested a role for airborne transmission when discussing the findings in context.^17^ We assume for this analysis that the transmission event occurred via the airborne mode, and we evaluated this assumption to the extent possible by comparing airborne viral exposure—a function of the level of rebreathed air and the rate of shedding into exhaled breath aerosols—between the single EG where transmission occurred and the other 12 EGs without transmission.

We applied the rebreathed air equation to the EMIT transmission trial data to estimate an airborne influenza infectious dose generation rate *q* to give rise to the observed 1.33% SAR. The relationship between *q* and the observed rate of RNA copy shedding into fine particle exhaled breath aerosols/h, *V* is defined by Equation 2,

(2) $$q=\frac{V}{\sigma }$$

where *σ* is the number of RNA copies per quantum (ID_63_) and represents the difference between estimated RNA copy airborne exposure, and the viral RNA quantity that reaches a vulnerable locus in the respiratory tract and evades the host immune system. Substituting for *q* gives Equation 3, which can be applied to the trial data to evaluate *σ*.

(3) $$P=\frac{D}{S}=1-\exp\left(-\frac{\bar{f}IVt}{n\sigma }\right)$$

### 2.5 Data manipulation, analysis, and statistics

Data were cleaned and analyzed in R Studio (Rv3.6.1; R Development Core Team) and SAS Studio (Release 3.7 [Enterprise Edition], v9.4M6). The development of new equations to evaluate the relationship between *q* and aerosolized RNA copy exposure is described in Results. Empirical bootstraps with 10 000 samples were used (base R) to produce 95% confidence intervals for *q* and *σ*. Residual standard error for the cumulative viral exposure in each EG was computed by linear regression of inhalation exposure on EG.

## 3 RESULTS

### 3.1 Exposure to exhaled breath from infectious donors

**FIGURE 1:**
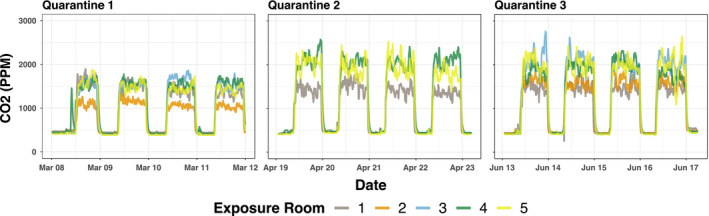
Observed CO_2_. Concentrations measured at 5‐min intervals over the entire course of the 4‐d exposure period

**FIGURE 2:**
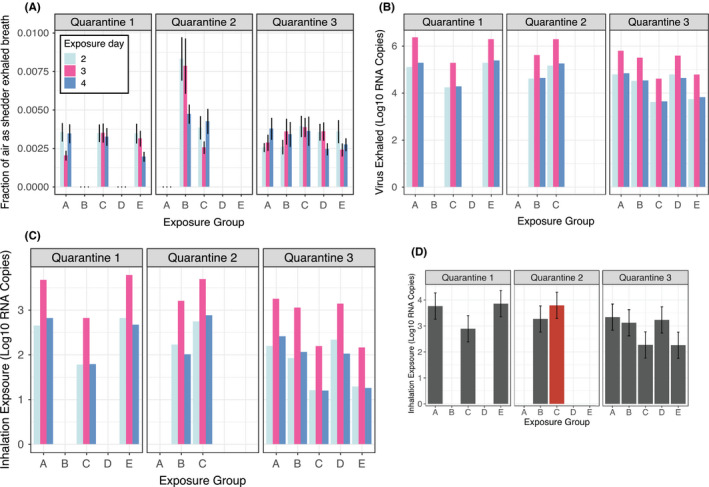
Exposure related to transmission risk. A, shows for each Recipient in each exposure group (EG), the fraction of inhaled air containing exhaled breath from Donors who shed detectable virus into fine aerosols (1 SD error bars). B, shows Donor shedding in each EG by Day, and C and D, show Recipient exposure to viral RNA aerosols in each EG by Day, and cumulatively, respectively. The single transmission event occurred in Quarantine 2 EG C, depicted in red (residual standard error bars in plot D)

Exposure room CO_2_ concentrations varied (Figure 1); however, the fraction of exhaled breath from viral shedders in each room $\frac{\bar{f}I}{n}$ was balanced by the rotation of Exposure Groups (EGs) to different rooms (Figure 2A). Compared with other EGs, the fraction was about double in Quarantine 2 EG B because there were two aerosol shedding Donors vs a maximum of one in the other EGs. Three EGs had no Donors with observed viral shedding into exhaled breath aerosols.

### 3.2 Viral RNA shed into exhaled breath aerosols

Measured fine aerosol shedding rates enabled conversion of Donor‐exhaled breath exposure to viral inhalation exposure. Of the 42 infected Donors, three gave samples on one day, 33 on two, four on three, and two on four (20, 25, 24, and 20 exhaled breath samples collected on Study Days 1, 2, 3, and 4,, respectively). 26% (11/42) of infected Donors had detectable influenza A viral RNA fine aerosol shedding (N = 14 detectable samples). 14% (6/42) of infected Donors had detectable coarse aerosol shedding (N = 6 detectable samples). All Donors who shed into coarse aerosol also shed into fine. A single Donor who did not meet infection criteria but had evidence of airborne shedding was excluded (Appendix S2).

Adjusting for quantification limit censorship and Donor‐Days without measurement, 33 RNA copies per hour (CPH) Donor‐Day values (imputed or observed for 11 Donor who ever shed into fine aerosols for Days 2‐4) had adjusted geometric mean (GM) of 4.7E + 3 RNA CPH and range 2.6E + 2‐1.6E + 5 and geometric standard deviation (GSD) of 6.0 (Table 1 and Figure 2B, parameter estimates in Tables S1‐S4, and model diagnostic Figure S1). Adjusting for quantification limit censorship, the 14 detectable fine aerosol samples had GM 1.0E + 4 RNA CPH, with GSD 4.7, and range 2.0E + 3‐1.6E + 5 (Table S5, Appendix S3, Figure S2). Coarse aerosols were assumed to not contribute to airborne risk in this model given their higher settling velocity and the contribution to substantially less exhaled breath influenza RNA (Appendix S4).

**TABLE 1:** Fine particle aerosol shedding strength from detected and estimated samples

Quarantine	Exposure group	Day 2	Day 3	Day 4	Daily average^c^
1	A	8.8E + 3 (1, 2)	1.6E + 5 (1, 2)	1.3E + 4 (0, 2)	1.8E + 5 (2, 6)
	B^a^	ND (0, 2)	ND (0, 1)	ND (0, 2)	ND (0, 5)
	C	1.2E + 3 (0, 3)	1.3E + 4 (1, 2)	1.3E + 3 (0, 1)	1.6E + 4 (1, 6)
	D^a^	ND (0, 2)	ND (0, 2)	ND (0, 2)	ND (0, 6)
	E	1.4E + 4 (0, 2)	1.3E + 5 (1, 3)	1.6E + 4 (1, 3)	1.6E + 5 (2, 8)
2	A^a^	ND (0, 2)	ND (0, 4)	ND (0, 0)	ND (0, 6)
	B	2.7E + 3 (1, 4)	2.7E + 4 (1, 2)	2.9E + 3 (0, 1)	3.3E + 4 (2, 7)
	C^b^	9.8E + 3 (1, 4)	1.3E + 5 (1, 4)	1.2E + 4 (0, 3)	1.5E + 5 (2, 11)
3	A	4.1E + 3 (0, 2)	4.0E + 4 (0, 2)	4.3E + 3 (1, 2)	4.9E + 4 (1, 6)
	B	2.0E + 3 (1, 2)	2.0E + 4 (0, 1)	2.1E + 3 (0, 2)	2.4E + 4 (1, 5)
	C	2.6E + 2 (0, 2)	2.6E + 3 (1, 2)	2.8E + 2 (0, 2)	3.2E + 3 (1, 6)
	D	3.9E + 3 (1, 2)	2.5E + 4 (0, 2)	2.7E + 3 (0, 2)	3.2E + 4 (1, 6)
	E	3.4E + 2 (0, 2)	3.9E + 3 (1, 3)	4.1E + 2 (0, 2)	4.6E + 3 (1, 7)

Note

RNA copies/h shed into fine particle exhaled breath aerosols by Day‐EG (*V_jk_*), by EG (*V_k_*) from observed and imputed samples (number samples with at least 1 detectable qRT‐PCR replicate, number samples tested). Details about imputed sample estimation in the “Quantifying viral shedding in exhaled breath” subsection of the Section 2.

^a^ EGs with no Donors shedding any fine aerosols with at least one qRT‐PCR replicate positive (ND, not detected = 0/2 qRT‐PCR replicates detected, or no sample collected).

^b^ EG with the transmission event.

^c^ Not time weighted.

### 3.3 Computation of *q*

The transmission trial represents a discrete exposure quantity for each of 75 susceptibles, where one became infected. By summing the probability of infection, *P* for each Recipient, we computed *q* for the trial as a whole. A similar approach was used to sum risk of transmission across multiple exposure periods between school children during a measles outbreak.^14^ We adjusted for the withdrawal of two Recipients before the end of Study Day 4. The withdrawal of a Recipient terminated the exposure for the withdrawn Recipient and splits the exposure in the EG for the remaining Recipients into partition *l* of EG *k*, on Study Day *j*, for Recipient *I* (Equation 4),

(4) $$R_{\text{total}}-R_{\text{infected}}=\sum_{\mathrm{ijkl}}\exp\left(-\frac{\bar{f}t_{\mathrm{ijkl}}I_{\mathrm{ijk}}q}{n_{\mathrm{ijkl}}}\right)$$

where *R*_total_ is 75, the total number of Recipients in the study, and *R*_infected_ is one, the total number of secondary cases. Solving by minimizing the difference between the sides of the equation and taking *I* as the number of Donors ever observed during the exposure period to have shed into fine aerosols (N = 11) resulted in *q* = 0.11 (95% CI 0.088, 0.12)/h. Using *I* as the total number of infected Donors (including non‐shedders; N = 42) yielded *q* = 0.029 (0.027, 0.030)/h. Theoretically raising or lowering CO_2_ by 10% altered *q* values by no more than a factor of 0.85 (Table S6 and Figure S3). Doubling the number of transmission events (ie, from 1 to 2) would have produced *q* = 0.21 (0.18, 0.24).

### 3.4 Computation of *σ*

We adopted a cumulative viral exposure term for each Recipient *r*_i_ over all Days, EGs, and Recipient withdrawal partitions (Equation 5).

(5) $$r_{i}=\sum_{{\left(jkl\right)}_{i}}\frac{\bar{f}t_{{\left(jkl\right)}_{i}}V_{{\left(jk\right)}_{i}}}{n_{{\left(jkl\right)}_{i}}}$$

The EG where the transmission event occurred (Quarantine 2, EG C) had among the highest exposure to viral RNA (Figure 2C,D and Table 2). We solved for *σ*, the viral RNA exposure from exhaled breath per quantum (Equation 6),

(6) $$R_{\text{total}}-R_{\text{infected}}=\sum_{i}\exp\left(-\frac{r_{i}}{\sigma }\right)$$

which gave *σ* = 1.4E + 5 RNA copies/quantum (95% CI 9.9E + 4, 1.8E + 5). Theoretically raising or lowering CO_2_ by 10% altered *σ* by no more than a factor of 0.86 (Table S6; Figure S3).

**TABLE 2:** Total fine particle aerosol viral exposure

Quarantine	Exposure group	Total aerosol viral exposure (log10 RNA copies per Recipient)
1	A	3.8
	B^a^	0.0
	C	2.9
	D^a^	0.0
	E	3.9
2	A^a^	0.0
	B	3.3
	C^b^	3.8
3	A	3.3
	B	3.1
	C	2.3
	D	3.2
	E	2.3

Note

Total estimated exposure over the 4‐d exposure period.

^a^ EGs with no Donors shedding any fine aerosols with at least one qRT‐PCR replicate positive.

^b^ EG with the transmission event.

According to this model, the maximum fine aerosol shedder among the experimentally infected Donors barely produced a single quantum/h into aerosols on the highest shedding day, while a Donor shedding at the GM generated 0.03 quanta/h. Given that Yan et al^4^ reported a correlation between fluorescent focus units (FFU) and fine aerosol RNA copies (*r* = .34, *P* < .0001), in a population of symptomatic influenza cases, we applied their ratio of RNA copies to FFU for influenza virus of approximately 1.0E + 3 and converted *σ* to 1.4E + 2 FFU/quantum, a rough estimate of virus particles/quantum. We applied the *q* and *σ* to several airborne exposure scenarios.

### 3.5 NIOSH sampler aerosol detection limit

NIOSH bioaerosol samplers were deployed in the EMIT exposure rooms for up to 3 hours sampling at a flow rate of 3.5 L/min.^22^ No viral RNA was detected from any of the samples immersed in 1mL UTM. Given an average human breathing rate *P* of 8 L/min, the samplers collected air at 44% that of human inhalation. The maximum collected by the sampler would have been 5.5E + 2 RNA copies/mL, which is below the limit of quantification for the assay given the dilution factors from nucleic acid extraction and qRT‐PCR protocols.^4^ Therefore, the lack of RNA detection in the NIOSH samplers is consistent with the low quantities of RNA estimated in the exposure room air.

### 3.6 Applying *q* to influenza challenge‐transmission “proof‐of‐concept” study

A proof‐of‐concept study prior to EMIT demonstrated the feasibility of human transmission following nasal inoculation of seronegative volunteers.^18^ We modified the Wells‐Riley equation given an 8.3% SAR (1/12 seronegative Recipients) with a standard pulmonary ventilation rate of 8 L/min,^15^ to estimate the ventilation rate in the exposure rooms (Equation 7). We assumed infected Donors generated *q* at the same rates as in the EMIT trial.

(7) $$R_{{\text{total}}_{\text{seronegative}}}-R_{\text{infected}}=\sum_{i_{\text{seronegative}}k}\exp\left(-\frac{I_{i_{\text{seronegative}}k}qpt}{Q}\right)$$

Solving for *q* = 0.11/h and 0.029/h gave estimated exposure room ventilation flow rates of *Q* = 6.7 L/s and *Q* = 1.8 L/s, respectively. Although we do not have knowledge of the exposure room ventilation, it is possible to evaluate the realism of these values. The study was conducted in hotel rooms with 42.3 m^3^ (4.6 m × 4.0 m × 2.3 m ceiling height) measured volume. Rooms were ventilated through infiltration and intermittent bathroom extract fan, which operated with the light switch. Windows were closed throughout the study and thermal comfort was maintained by an air conditioner that recirculated room air but provided no supply air. Using these parameters, the overall air change rate in the rooms can be estimated as 0.15 air changes per hour (ACH) based on the lower value of *Q*, or 0.57 ACH based on the higher value. The room is clearly poorly ventilated with either estimate; however, the lower air change rate is highly unlikely for an upper floor of a commercial building,^23^ suggesting that the value of *q* = 0.11/h from the EMIT trial is a much more realistic value for the proof‐of‐concept study. The higher *q*—with aerosol shedders only (*I* = 11)—is consistent with the proof‐of‐concept study protocol in selecting Donors who were likely to have been shedding for each Study Day of exposure.

### 3.7 Applying *σ* to an airplane influenza outbreak

Moser et al, 1979 reported an influenza A/H3 outbreak in a Boeing 737 attributed to exposure to an intensely ill passenger during a 4.5‐hour ground delay with no operational, mechanical ventilation. Ventilation rates were unmeasured. Given the SAR of 72% among 54 people on board, well above the average reproduction ratio for influenza, it is plausible that the individual on board was a super shedder. The 95th percentile of RNA shed in exhaled breath fine aerosols by the symptomatic influenza cases described in Yan et al^4^ was 7.4E + 6 CPH on day 1 post onset (the closest measure to illness onset available). Assuming the super shedder on the Boeing 737 was shedding at the same rate and applying *σ* = 1.4E + 5 and Equation 2 (Section 2), *q* = 53/h for the airplane scenario. This estimate is consistent with those computed by Rudnick and Milton, *q* = 79 or *q* = 128/h, using estimated outdoor air exchange rates of 0.1/h and 0.5/h, respectively.^15^

### 3.8 Estimating *q* for a population of symptomatic, naturally infected cases

Yan et al^4^reported influenza viral shedding of 218 half‐hour exhaled breath samples among 142 symptomatic individuals from a population of mostly young adults in the University of Maryland community. Taking into account samples assumed to contain RNA in quantities below the limit of detection, the adjusted geometric mean RNA recovered from exhaled breath fine particle aerosols was 2.4E + 4 (95% CI 1.4E + 4, 2.8E + 4) CPH. Applying Equation 2 and *σ* = 1.4E + 5 RNA copies/quantum yields *q* = 0.17 quanta/h (95% CI 0.10, 0.27) for the symptomatic population. Meanwhile, a symptomatic, naturally infected case shedding at the maximum daily rate into aerosols produced 630 quanta/h. The influenza cases from Yan et al^4^ were selected from symptomatic individuals who presented to the University health center or directly to the study within the first 3 Days of illness and were febrile > 37.8°C with cough or sore throat or had a positive QuickVue rapid test. This population may not be representative of the viral shedding that might be expected from a broader population of influenza cases given that many cases are mildly or asymptomatic.^24^, ^25^ If symptom severity is positively correlated with shedding strength, then the *q* computed for Yan et al may over‐estimate the *q* for the broader influenza‐infected population, which may be better represented by the EMIT trial's mostly mildly symptomatic Donors.

### 3.9 Estimating airborne transmission risk for roommates

**FIGURE 3:**
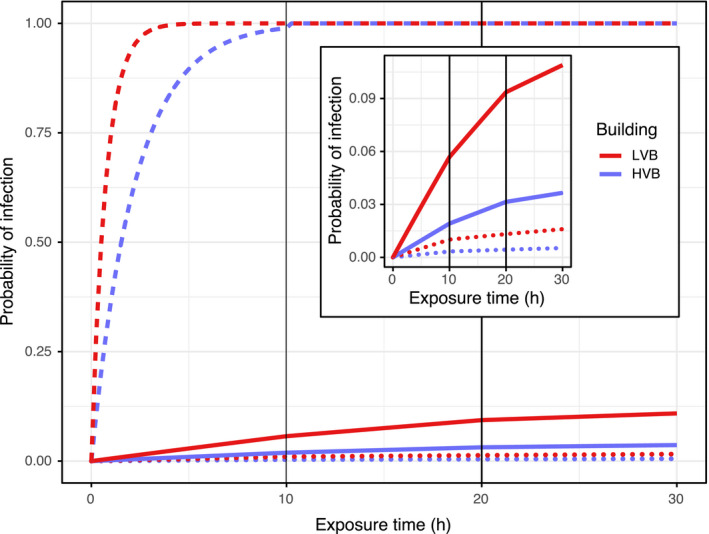
Probability of infection for a theoretical, susceptible roommate in LVB (low ventilated building) and HVB (high ventilated building) dormitory rooms for 3 days, where each day is assumed to have 10 h of exposure; solid lines represent median fine aerosol shedding rates (RNA copies/h) dots represent 10th percentile, and dashes represent 90th percentile, from a symptomatic population of influenza cases^4^

The computed *σ* can be used to estimate probability of airborne transmission in a variety of non‐experimental settings using the Wells‐Riley or rebreathed air equations (Equations S1 and S2). We used hypothetical scenarios in a “high” and “low” ventilated dormitory, each with one infected and one susceptible roommate, given measured airborne viral shedding rates and modeled dormitory room ventilation of 12.1 (1 ACH) and 4.0 L/s (0.3 ACH) in a high and low ventilated dormitory, respectively, based on an analysis of indoor air ventilation (Appendix S5).^26^ Reanalyzing the fine aerosol shedding rate for the 142 influenza cases using the final adjusted model from Yan et al^4^ gives median 5.8E + 6, 1.2E + 6, and 5.4E + 5 for days post onset 1, 2, and 3, respectively. Assuming 10 hours of exposure per day between roommates (about eight during the night plus two during the day) and the declining aerosol shedding rates over 3 days (a single shedding rate used per day), the modeled probability of transmission is displayed for roommates of a shedder at the by‐day 10th, 50th, and 90th percentiles in the “high” and “low” ventilated dorm rooms (Figure 3). Risk is more than double in the low compared with the high ventilated dorm room for a shedder at the median or 10th percentile. A supershedder with 90th percentile CPH per exposure day posed substantially greater risk in the “high” vs “low” ventilated dorm rooms up to a few hours of exposure, but risk approached 100% for both dorms by the end of 10‐hour exposure, illustrating potential limits of ventilation for reducing transmission in the presence of a supershedder.

## 4 DISCUSSION

We applied the rebreathed air equation with measured viral shedding and CO_2_ concentrations from the largest, human challenge‐transmission trial (N = 52 experimentally infected viral Donors, N = 75 Recipients) to compute an airborne influenza quantum (ID_63_) generation rate of 0.11 (95% CI 0.088, 0.12)/h for experimentally infected viral aerosol shedders. To support clarity about the relationship between infectious quantum and airborne ID_63_ (the latter more widely used in medical literature), we refer readers to work by Wells,^13^ and by Rudnick and Milton.^15^ We also quantified a quantum of airborne infection in terms of RNA copies. Our estimates are consistent with a proof‐of‐concept challenge‐transmission study, and an airliner outbreak. In the context of a hypothetical dormitory exposure scenario with symptomatic influenza cases, findings demonstrate supershedders and indoor ventilation as potential drivers of airborne transmission.

Alford et al challenged humans with metered airborne viral inhalation exposure, demonstrating an airborne infectious dose of about 1 (TCID_50_).^7^ Differences in culture methodology aside, it is unsurprising that our estimated *σ* of 1.4E + 2 FFU/quantum was higher given some variation in what FFU means for quantitating infectious virus, and because *σ* represents the inhalation exposure to contaminated air in the breathing zone as opposed to direct dosage via mouthpiece inhalation as done by Alford and colleagues. Our findings of low quanta generation rate are consistent with reports of low reproductive number and the observation of larger outbreaks associated with prolonged exposure.^19^, ^27^ Compared with our *σ* of 1.4E + 5 RNA copies/quantum, the fine aerosol viral shedding rate from the 26% of infected Donors with detectable fine aerosol shedding was small, GM 4.7E + 3 and range 2.6E + 2‐1.6E + 5 CPH. The observed maximum aerosol shedding rate barely reached a single quantum/h.

Because the single transmission event in the EMIT trial occurred in a Control Recipient, the mode of transmission is unclear; however, some evidence supports the airborne transmission assumption. The EMIT trial resulted in 1.33% SAR, although a proof‐of‐concept experimental human influenza challenge‐transmission trial using half the exposure time and fewer than half the infectious Donors gave SAR 8.3%, using the infection criteria of the former.^18^ Compared with proof‐of‐concept, after increasing the magnitude and duration of exposure, the follow‐on transmission trial was expected to produce a 16% SAR. Thus, the observed SAR of 1.33% was much lower than expected under identical study conditions (*P* < .001).^17^ Nguyen‐Van‐Tam and colleagues point out that the main difference between these studies was likely the room ventilation.^17^ The proof‐of‐concept study was performed in hotel rooms, with relatively little ventilation compared with the follow‐on trial in a controlled environment. That the SAR did not increase between the proof‐of‐concept and follow‐on trials, yet the magnitude and duration of direct and indirect contact between infectious and susceptible volunteers more than doubled, and the air exchange rate likely increased—which would promote dilution of infectious airborne particles—supports the interpretation that airborne particles and not contact or large droplets drove transmission in this model. The finding that the single transmission event occurred in a Recipient with one of the highest levels of airborne exposure and that volunteers without viral any aerosol exposure remained uninfected further supports the interpretation that transmission was via fine aerosols. Despite this, the low SAR limits the reliability of the estimated *q* and confidence bounds. Coarse aerosols are less likely to contribute to airborne transmission because they settle quickly and contributed much fewer RNA CPH. That a transmission event occurred in just one of the three EGs with the highest airborne exposure and no other EGs is consistent with the finding of low overall transmission risk in the EMIT quarantine model, and could also reflect the stochastic nature of infection events.

In this study, the two main factors contributing to transmission risk were the rate of fine aerosol shedding from infectious cases, and the level of rebreathed air in the indoor exposure space governed by the air exchange rate. That a typical, symptomatic, naturally infected influenza case in an H3 predominant season^4^ generated 0.17 quanta/h while one shedding at the maximum rate produced 630 quanta/h suggests a role for supershedders in airborne influenza transmission.

The hypothesis that supershedders drive transmission is supported by application of the Wells‐Riley equation (Equation S1) to estimate transmission risk between roommates in dormitory rooms with modeled ventilation rates (Figure 3). Transmission risk attributable to a roommate shedding at the median almost reached 10% after 3 days in a low ventilated dormitory, while that attributable to a roommate at the 90th percentile reached 100% within the first day for both dormitories (about 6 hours faster in the low ventilated compared with the high ventilated dormitory). For the symptomatic population described by Yan and colleagues,^4^ a roommate shedding at the 90th percentile produces the equivalent exhaled virus of approximately 80‐250 median shedders, depending on the day post symptom onset. A shedder at the 95th percentile produces the equivalent of approximately 300‐1100 median shedders. However, it is still uncertain to what extent transmission is driven by supershedders vs average shedders that may be contaminating low ventilated spaces. Phylogenetic studies could evaluate the theory that a minority of viral shedders are responsible for the bulk of transmission using a previously described analytical framework.^28^ Studies that can refine predictors of high‐level aerosol shedding, as done by Yan and colleagues, or can refine predictors of transmission related to indoor environments are of great importance to new research efforts and population level disease prevention efforts.

Studies of influenza transmission in households have reported SARs of 8% and 21%.^29^, ^30^ Analysis of these household trials—which used hand hygiene and facemask interventions to control for transmission mode—reported that airborne influenza could be responsible for about half of influenza transmission events and that interventions to interrupt contact and large droplet modes may not reduce overall risk, but rather shift transmission mode.^12^ Thus, the household study SARs due to airborne risk alone may be about 4%‐10%, which is very similar to that observed in the dormitory scenario with typical shedders (Figure 3). An analysis of a separate household cohort found that among 52 sample pairs between primary and potential secondary household transmission cases with sequence data of sufficient quality, 47 (90%) were considered phylogenetically supported transmission events.^31^ This lends credence to the assumption that household transmission events in the aforementioned household studies were between household contacts. Despite this, it may be a minority of infections that are acquired from household contacts in the residential setting. Reanalysis of household transmission data described in McCrone et al,^31^ assuming one case in each household came from an outside source, shows 72% of influenza cases originated from sources outside the household. This is consistent with literature on TB transmission in high‐burden settings with risk attributable to non‐household resident sources estimated at 65%‐81% in rural and urban settings globally.^32^, ^33^, ^34^, ^35^ This may suggest shorter‐term shared air exposure to supershedders as important for TB transmission, a hypothesis which may also hold for influenza transmission.

The experimental design of the EMIT challenge‐transmission trial enabled a comprehensive airborne exposure assessment, but contained features that limited generalizability. Volunteers were all healthy, young adults above age 18, were selected with low levels of preexisting H3 antibodies, and temperature and humidity were selected to optimize environmental conditions favorable for influenza transmission.^36^, ^37^ Psychosocial stress was not observed despite its importance in infection susceptibility.^38^ Comparison of wild‐type reference virus to the prepared A/Wisconsin/67/2005 (H3N2) used for inoculation showed partial adaptation to laboratory culture environments yet conservation of a fixed variant in the HA gene, suggesting that the laboratory‐prepared virus was not likely to be the cause of the low SAR.^17^, ^39^ There was minimal heterogeneity in host immunity, viral, coinfection, contact exposure networks, and temperature and humidity. Addressing the effect of these factors on transmission risk by airborne and other modes is required to translate findings into actionable population infection prevention. The *σ* from this model can be applied to high‐risk and public settings such as public transportation and hospital waiting areas, in which ambient airborne viral concentrations could be measured to translate inhalation exposure into estimated inhalation ID_63_ values. The *q* paired with measured or theoretical ventilation information enables the estimation of airborne risk in indoor settings should an infectious case enter the indoor environment. The influence of super shedders vs typical airborne shedders on risk to exposed susceptibles in realistic, theoretical scenarios can be modeled to inform population health prevention measures. To improve generalizability of these findings, perhaps at the expense of feasibility, a hybrid challenge study could be used, recruiting naturally infected cases as Donors and a demographically and immunologically diverse population as Recipients. Alternatively, the isolation of H1N1 variants unique to lower respiratory tract compared with the nasal mucosa points toward the possibility of transmission mode tracing through sequencing samples from community exposure networks.^40^

In conclusion, we used the largest, human challenge influenza transmission study to estimate a reasonable airborne infectious dose generation rate *q* and RNA copies per infectious dose *σ*. We presented a powerful and feasible methodology for estimating these parameters and predicting transmission risk given measurements of the source strength of virus shed into exhaled breath and indoor air characteristics. Our model shows that *q* for experimentally infected Donors with A/WI virus in the controlled, challenge environment was low, consistent with expectations, and that typical, experimentally infected aerosol shedders and naturally infected community cases generated few infectious doses/h, however cases who shed the most virus generated several hundred infectious doses/h. Our application of these findings to a university dormitory transmission scenario showed that airborne risk in the presence of an average shedder could be substantially mitigated by increased ventilation, but perhaps not in the presence of a supershedder. This work highlights the potential for airborne transmission as a driver of epidemics and underscores the need to better characterize drivers of infectious viral shedding and the effect of built environments and exposure controls and their roles in transmission risk, population surveillance, and epidemic and pandemic prevention and readiness.

## CONFLICTS OF INTEREST

There are no conflicts to declare.

## AUTHOR CONTRIBUTION

**Paul Jacob Bueno de Mesquita:** Conceptualization (equal); Data curation (lead); Formal analysis (lead); Methodology (equal); Visualization (lead); Writing‐original draft (lead); Writing‐review & editing (lead). **Catherine J. Noakes:** Formal analysis (supporting); Writing‐review & editing (supporting). **Don K. Milton:** Conceptualization (equal); Formal analysis (equal); Methodology (equal); Supervision (lead); Writing‐review & editing (equal).

## Supporting information

Supplementary Material

## Data Availability

Data required for reproduction of analyses are available upon request. Scripts to reproduce analyses are available at: https://gitlab.com/jacobbueno/virus_in_exhaled_breath.
