# Supplementary material for: Quantitative aerobiologic analysis of an influenza human challenge‐transmission trial
Source: Indoor Air. 2020 Jun 15;30(6):1189–98. doi: 10.1111/ina.12701 (PMC7687273; doi:10.1111/ina.12701)
Supplement: Supplementary file 1 — Supplementary Material [file INA-30-1189-s001.docx]

**SUPPLEMENTARY INFORMATION (SI)**

**SI for**

Quantitative aerobiologic analysis of an influenza human challenge-transmission trial

P. Jacob Bueno de Mesquita, Catherine Noakes, and Donald K. Milton

**SI contents:**

- EMIT Consortium Team Members
- Appendix 1. Wells-Riley and rebreathed-air equations
- Appendix 2. Note about one volunteer excluded as an infected or aerosol shedding Donor
- Appendix 3. Tobit regression model parameters and diagnostics
  - Tables S1-S4
- Appendix 4. Assumption about low risk of large droplet transmission
- Appendix 5. Indoor CO_2_ monitoring for EMIT and in University of Maryland dormitories
- Figures S1-S3
- Tables S5-S6
- SI References

**EMIT Consortium Team Members**

EMIT team members were: Walt Adamson, Blanca Beato-Arribas, Werner Bischoff, William Booth, Simon Cauchemez, Sheryl Ehrman, Joanne Enstone, Neil Ferguson, John Forni, Anthony Gilbert, Michael Grantham, Lisa Grohskopf, Andrew Hayward, Michael Hewitt, Ashley Kang, Ben Killingley, Robert Lambkin-Williams, Alex Mann, Donald Milton, Jonathan Nguyen-Van-Tam, Catherine Noakes, John Oxford, Massimo Palmarini, Jovan Pantelic, and Jennifer Wang. The Scientific Advisory Board members were: Allan Bennett, Ben Cowling, Arnold Monto, and Raymond Tellier.

**Appendix 1. Wells-Riley and rebreathed-air equations**

The Wells-Riley equation has been used in numerous studies to estimate transmission risk for Tuberculosis^1-3^. The Wells-Riley equation for estimating the probability of indoor airborne transmission requires assumptions of well-mixed air space and steady-state conditions and is defined as:

$P=\frac{D}{S}= 1-exp(-\frac{Ipqt}{Q})$ (S1)

with *P* probability of infection for *S* exposed susceptibles, *D* secondary infections, *I* infectors in their infectious stage (i.e., when they are emitting virus), *p* breathing rate per indoor occupant (L/s), *q* quantum generation rate from each *I* (quanta/h), *t* exposure time (h), and *Q* outdoor air supply rate (L/s)^4^.

Rudnick and Milton described *q* as the “average infectious source strength of infected individuals”^5^. They emphasized a) that a quantum is not an organism but rather a dose that reflects the stochasticity of airborne contagion, and b) that an infectious dose may not be greater than a single organism that reaches a vulnerable locus. Rudnick and Milton’s ‘rebreathed-air’ adaptation of the Wells-Riley equation directly estimates inhalation exposure to concentrations of airborne contaminants in exhaled breath using CO_2_ as a marker of exhaled breath concentration^5^. It is defined by:

$P=\frac{D}{S}= 1-exp(-\frac{\overline{f}Iqt}{n})$ (S2)

where $\overline{f}$ is the time-weighted average fraction of indoor air that is exhaled breath with *n* individuals in the room contributing CO_2_. Thus, $\frac{I}{n}$ equals the fraction of the $\overline{f}$, or the ‘rebreathed fraction,’ from an infectious individual.

**Appendix 2. Note about one volunteer excluded as an infected or aerosol shedding Donor**

There was an instance where a Donor (subject 109) had one replicate qRT-PCR detected for fine aerosols but no positive swabs and no indication of seroconversion, so we did not count this as a true infection and did not include their aerosols samples as positive below LOD in the final analysis.

**Appendix 3. Tobit regression model parameters and diagnostics**

1. Tobit model using 19 samples (N=10, N=4, and N=5, where two, one, and zero out of two qRT-PCR replicates were detectable, respectively) from a total of 11 Donors who ever shed into fine particle aerosols. Model: fixed effects of cough and Study Day with random effect of person to predict fine aerosol shedding RNA CPH. Parameter estimates from this model were applied to the additional 14 samples where no samples were collected to yield viral shedding rate estimates for 33 total samples (one sample for each Study Day 2-4 for each of 11 Donors who ever had detectable aerosol shedding).

| **Table S1. Full Tobit model fit statistics** | |
| --- | --- |
| **-2 Log Likelihood** | 29.9 |
| **AIC** | 41.9 |
| **AICC** | 44.6 |
| **BIC** | 44.3 |

| **Table S2. Full Tobit model parameter estimates** | | | | | | | | |
| --- | --- | --- | --- | --- | --- | --- | --- | --- |
| **Parameter** | **Estimate** | **Standard Error** | **DF** | **t Value** | **Pr > \|t\|** | **95% Confidence Limits** | | **Gradient** |
| **Random effect of person intercept** | 0.3568 | 0.1636 | 10 | 2.18 | 0.0542 | -0.00772 | 0.7214 | -0.00010 |
| **Random effect of person** | 0.04104 | 0.01532 | 10 | 2.68 | 0.0231 | 0.006913 | 0.07517 | 0.001449 |
| **Intercept** | 2.9801 | 0.2074 | 10 | 14.37 | <.0001 | 2.5180 | 3.4421 | -0.00001 |
| **Cough score (daily average)** | 0.08768 | 0.2605 | 10 | 0.34 | 0.7434 | -0.4928 | 0.6681 | 0.000129 |
| **Study Day 3 vs Study Day 2** | 0.9946 | 0.1825 | 10 | 5.45 | 0.0003 | 0.5880 | 1.4013 | 0.000196 |
| **Study Day 4 vs Study Day 2** | 0.02316 | 0.1773 | 10 | 0.13 | 0.8987 | -0.3720 | 0.4183 | 0.000080 |

1. Tobit model using 14 samples (N=10 and four, where two and one out of two qRT-PCR duplicates were detectable, respectively) from a total of 11 Donors who ever shed into fine particle aerosols. Model: fixed effects of cough and Study Day with random effect of person to predict fine aerosol shedding RNA CPH.

| **Table S3. Detectable samples model fit statistics** | |
| --- | --- |
| **-2 Log Likelihood** | 27.5 |
| **AIC** | 39.5 |
| **AICC** | 43.5 |
| **BIC** | 41.9 |

| **Table S4. Detectable samples parameter estimates** | | | | | | | | |
| --- | --- | --- | --- | --- | --- | --- | --- | --- |
| **Parameter** | **Estimate** | **Standard Error** | **DF** | **t Value** | **Pr > \|t\|** | **95% Confidence Limits** | | **Gradient** |
| **Random effect of person intercept** | 0.3534 | 0.1626 | 10 | 2.17 | 0.0549 | -0.00896 | 0.7157 | 2.357E-6 |
| **Random effect of person** | 0.04063 | 0.01549 | 10 | 2.62 | 0.0255 | 0.006107 | 0.07515 | 0.000011 |
| **Intercept** | 2.9345 | 0.2091 | 10 | 14.03 | <.0001 | 2.4685 | 3.4005 | 1.893E-6 |
| **Cough score (daily average)** | 0.03770 | 0.2624 | 10 | 0.14 | 0.8886 | -0.5469 | 0.6223 | -0.00002 |
| **Study Day 3 vs Study Day 2** | 1.0832 | 0.1939 | 10 | 5.59 | 0.0002 | 0.6513 | 1.5151 | -0.00002 |
| **Study Day 4 vs Study Day 2** | 0.2448 | 0.2353 | 10 | 1.04 | 0.3225 | -0.2794 | 0.7690 | 0.000010 |

**Appendix 4. Assumption about low risk of large droplet transmission**

While fine particle aerosols are involved in airborne exposure, coarse particle aerosols represent larger droplets, and could potentially initiate infection should they land directly on a mucosal membrane of a susceptible or contaminate a fomite that is handled by a susceptible. They settle to the ground relatively quickly. There were only two coarse exhaled breath aerosol samples with both detectable replicates and their replicate averages were 5.6E+4 and 4.2E+3 RNA CPH, from Donors in Quarantine 1 EG E and Quarantine 3 EG B, respectively. There were four other positive coarse aerosol sample replicates that ranged from 2.6E+3 to 6.8E+4 RNA CPH. Virus contained in coarse aerosols was assumed to not contribute to transmission risk.

**Appendix 5. Indoor CO_2_ monitoring for EMIT and in University of Maryland dormitories**

**EMIT quarantine facility**

**Overview.** Environmental monitoring in the EMIT trial was carried out using calibrated wireless Radio-Tech sensors for CO_2_ (Eco2, +/- 30ppm) and temperature and humidity (Ecosense, <0.3^o^C, <3%RH) with readings in each EG taken every 5 minutes during the all Quarantine exposure. The arithmetic means (standard deviations) for background CO_2_ were 420 (25), 435 (20), and 420 ppm (10) for EMIT Quarantines 1, 2, and 3, respectively. Background levels were computed as the average CO_2_ concentrations observed between 02:00 and 03:00 hours (when rooms were unoccupied) in each exposure room during the four nights following daytime exposure events. The integrated exposure to exhaled breath over time in an indoor space, rebreathed fraction $\overline{f}$, was computed for each EG by integrating over the CO_2_ concentrations measured in the rooms over the four days of exposure (i.e., Study Days 1-4) after subtracting background CO_2_ levels and dividing by the constant CO_2_ concentration in exhaled breath, estimated as 3.8E+4 ppm.

To test the assumption that the exposure rooms were well mixed spaces, tracer gas studies were conducted in one of the exposure rooms. CO_2_ sensors in the corners and center of the room followed similar patterns in CO_2_ concentration levels that reflected CO_2_ releases into the rooms. These finding were robust to the opening and closing of the door.

**Environmental monitoring (excerpt from report prepared by BSRIA).**

*The environmental conditions were selected by the EMIT team such that they would create proper conditions for the virus to propagate, but at the same time avoiding undue discomfort from the temperature and humidity conditions whilst ensuring CO2 levels were kept within acceptable bounds. The design and preparatory works by BSRIA including pre-conditions of the exposure rooms immediately before occupants were introduced producing stable conditions across the five exposure rooms on any given Study Day and from quarantine period to quarantine period. Checks of ventilation rate using tracer gas after each quarantine period were among the techniques conducted to demonstrate uniformity and consistency. A data logging system was installed with two roles. Data was automatically sent at intervals to the “Cloud” to ensure the minimum reporting requirements for a clinical trial were met. The second role was to provide near live feedback to the BSRIA team present during the exposure events. BSRIA provided regular summaries including trend analysis throughout the exposure events to the Chief Investigator and the Retroscreen [currently hVIVO] clinical trials team ultimately responsible for execution of the quarantine periods.*

*In summary, BSRIA was able to ensure that the indoor environmental conditions during the exposure events met the requested environmental ranges. The preparation and pre-commissioning led by BSRIA established confidence that the environmental conditions could be helped throughout each of the sixteen-hour exposure events. This confidence was fully demonstrated. Data was collected from each room with very few missing values. Calibration of the sensors was conducted after the first quarantine and after the third quarantine and showed no significant drift. The database … contains both the raw data and the corrected data for use by other EMIT researchers.*

**University of Maryland dormitories**

University of Maryland dormitory ventilation surveillance including CO_2_ monitoring and calibration, pressure differentials, and CONTAM multi-zone modelling during the academic term 2017-2018 is described elsewhere^24^. Sensors (HOBO MX1102 Data Loggers) were placed high on walls and near doors so to avoid a directly plume of CO2 from dormitory room occupants. Background CO_2_ was observed from sensors placed outside the dormitories. Most of the rooms were two-person occupancy, with some single-, triple-, and quadruple-person occupancy rooms.

**Fig. S1.** Depicts observed minus Tobit model expected values for all 33 sample values (three Study Days for each 11 Donors who ever shed detectable qRT-PCR virus into fine aerosol).

****Fig. S2*.*** Depicts log_10_ RNA CPH shed into exhaled breath fine particle aerosol by Study Day, for samples with at least one detectable qRT-PCR replicate (n=14) and for all samples with at least one detected qRT-PCR replicate (left panel) in addition to samples below detection limit or unobserved with Tobit estimated shedding rates among ever-aerosol shedders (n=33) (right panel); the boxes show the interquartile range (IQR) with a band to indicate the median, and whiskers extending to the highest and lowest data points within 1.5 IQR.

**Fig. S3.** Infectious quanta generation rate and sigma. Effect of indoor CO_2_ level changes on point estimates (95% CI).

**Table S5. Fine particle aerosol shedding strength from detected samples**

| **Quarantine** | **Exposure Group** | **Day 2** | **Day 3** | **Day 4** | **Daily Average**^§^ |
| --- | --- | --- | --- | --- | --- |
| 1 | A | 8.8E+3 (1, 2) | 1.6E+5 (1, 2) | ND (0, 2) | 5.6E+4 (2, 6) |
|  | B^†^ | ND (0, 2) | ND (0, 1) | ND (0, 2) | ND (0, 5) |
|  | C | ND (0, 3) | 1.3E+4 (1, 2) | ND (0, 1) | 4.4E+3 (1, 6) |
|  | D^†^ | ND (0, 2) | ND (0, 2) | ND (0, 2) | ND (0, 6) |
|  | E | ND (0, 2) | 1.3E+5 (1, 3) | 1.6E+4 (1, 3) | 5.0E+4 (2, 8) |
| 2 | A^†^ | ND (0, 2) | ND (0, 4) | ND (0, 0) | ND (0, 6) |
|  | B | 2.4E+3 (1, 4) | 3.5E+3 (1, 2) | ND (0, 1) | 2.0E+3 (2, 7) |
|  | C**^‡^** | 9.8E+3 (1, 4) | 1.3E+5 (1, 4) | ND (0, 3) | 4.6E+4 (2, 11) |
| 3 | A | ND (0, 2) | ND (0, 2) | 4.3E+3 (1, 2) | 1.4E+3 (1, 6) |
|  | B | 2.0E+3 (1, 2) | ND (0, 1) | ND (0, 2) | 6.7E+2 (1, 5) |
|  | C | ND (0, 2) | 2.6E+3 (1, 2) | ND (0, 2) | 8.8E+2 (1, 6) |
|  | D | 3.9E+3 (1, 2) | ND (0, 2) | ND (0, 2) | 1.3E+3 (1, 6) |
|  | E | ND (0, 2) | 3.9E+3 (1, 3) | ND (0, 2) | 1.3E+3 (1, 7) |
| RNA copies shed into fine particle exhaled breath aerosols/h by Day-EG, $\bigvee_{jk}$, by EG, $\bigvee_{k}$ from samples with at least one detectable qRT-PCR replicate (number samples with at least 1 detectable qRT-PCR replicate, number samples tested). ND: not detected = 0/2 qRT-PCR replicates detected, or no sample collected.  ^†^EGs with no Donors observed to shed any fine aerosols with at least one qRT-PCR replicate positive. **^‡^**EG with the transmission event. ^§^Not time weighted; Assumed that ND=0. | | | | | |

**Table S6. Infectious quanta generation rate and** $\sigma$

| **Change in CO2** | ***q* _(all Donors)_** | ***q* _(aerosol shedders)_** | $\sigma$ |
| --- | --- | --- | --- |
| -50% | 0.089 (0.083, 0.094) | 0.32 (0.26, 0.36) | 2.4E+5 (1.7E+5, 3.0E+5) |
| -40% | 0.063 (0.059, 0.066) | 0.23 (0.19, 0.26) | 2.2E+5 (1.5E+5, 2.8E+5) |
| -30% | 0.048 (0.046, 0.051) | 0.18 (0.15, 0.2) | 2.0E+5 (1.4E+5, 2.5E+5) |
| -20% | 0.04 (0.037, 0.042) | 0.14 (0.12, 0.16) | 1.8E+5 (1.3E+5, 2.3E+5) |
| -10% | 0.033 (0.031, 0.035) | 0.12 (0.10, 0.14) | 1.6E+5 (1.1E+5, 2.0E+5) |
| **Observed** | **0.029 (0.027, 0.03)** | **0.11 (0.088, 0.12)** | **1.4E+5 (1.0E+5, 1.8E+5)** |
| +10% | 0.025 (0.024, 0.027) | 0.093 (0.077, 0.11) | 1.2E+5 (8.5E+4, 1.5E+5) |
| +20% | 0.023 (0.021, 0.024) | 0.084 (0.070, 0.094) | 1.0E+5 (7.2E+4, 1.3E+5) |
| +30% | 0.021 (0.019, 0.022) | 0.076 (0.063, 0.085) | 8.0E+4 (5.8E+4, 1.0E+5) |
| +40% | 0.019 (0.018, 0.02) | 0.069 (0.057, 0.078) | 6.0E+4 (4.4E+4, 7.6E+4) |
| +50% | 0.017 (0.016, 0.018) | 0.063 (0.053, 0.071) | 4.0E+4 (3.0E+4, 5.0E+4) |
| Effect of indoor CO_2_ level changes on point estimates (95% CI). | | | |

**SI References**

1. R. Wood, *et al.*, Tuberculosis transmission to young children in a South African community: modeling household and community infection risks. *Clin. Infect. Dis.* **51**, 401–408 (2010).
2. T. A. Yates, *et al.*, The transmission of Mycobacterium tuberculosis in high burden settings. *Lancet Infect Dis* **16**, 227–238 (2016).
3. J. G. Taylor, *et al.*, Measuring ventilation and modelling M. tuberculosis transmission in indoor congregate settings, rural KwaZulu-Natal. *The International Journal of Tuberculosis and Lung Disease* **20**, 1155–1161 (2016).
4. Riley, E. C., Murphy, G. & Riley, R. L. Airborne spread of measles in a suburban elementary school. *Am J Epidemiol* **107**, 421–432 (1978).
5. S. N. Rudnick, D. K. Milton, Risk of indoor airborne infection transmission estimated from carbon dioxide concentration. *Indoor Air* **13**, 237–245 (2003).
